# Supplementary material for: Regulating pharmacists as contraception providers: A qualitative study from Coastal Kenya on injectable contraception provision to youth
Source: PLoS One. 2019 Dec 19;14(12):e0226133. doi: 10.1371/journal.pone.0226133 (PMC6922368; doi:10.1371/journal.pone.0226133)
Supplement: S1 File — (DOCX) [file pone.0226133.s001.docx]

# Focus Group Discussion Guide

*Today we’re going to discuss what young people in this community think about contraceptives and where they go when they need it.*

**Warm-up**

- Tell me what “contraceptive” means to you?
  - Tell me the kinds of contraceptives you’ve heard of

**Myths and misinformation around contraception**

**Vignette:** XXX [name determined by FGD participants] is 21 and her boyfriend YYY [name determined by FGD participants] is 23. They have been dating for awhile and are thinking about using contraceptives. However, there are things about contraceptives that they have heard from friends and family members which make them uncertain.

What are some of the things which they may have heard?

*Ask participants to write down on sticky notes at least three things that XX and YY may have heard which would make them nervous. NoteTaker and Facilitator 3 will post these on the board, grouping together the similar ones. After they are all posted, moderator can ask:*

- *[read out the reasons listed on the board]:* Are there any additional reasons XX and YY may feel uncertain that you can think of?
- *[also probe on certain reasons that are vague or broad]*

**Where young people get contraceptives**

- Tell me about all the places in _____ (study site town), where a young person can get contraceptives? *(Facilitator 3 writes out a list)*
- Describe all the different kinds of young people you could find in your community. *(keep this short)*

*For each listed contraception source:*

- Describe the kind of young person who would go to a _______ if he/she needed contraceptives? *(Draw stick figure under each source name, probe on and label with identifiers: gender, marital status, etc)*

*Facilitator 3 stops drawing after question above*

- When would a young person choose to go to a _____ to get contraceptives?
  - [Note]: what kind of contraceptives are they getting
- Why would this young person choose to go to a ________ to get contraceptives over another source?
  - [Probe] What are the best qualities about ______ as a resource for contraceptives?
- What might other young people *dislike* about ________ as a resource for contraceptives?

**Qualities of ideal FP-dispensing in *non-service sources***

- What are the most important qualities a chemist or a shop needs to have for a young person to be comfortable obtaining contraceptives? [*Probe on person working vs the shop itself]*
- What could be some reasons why young people would not be comfortable going to chemists or shops?
  - What could be done to increase the comfort of young people who might not be comfortable going to chemists or shops?
- What **other** information and services would a young person needing contraceptives from a chemist or a shop also need?
  - [*be sure to probe on information AND services separately]*
- What could be done to make sure that young people can get the extra information and services (*that group mentioned in previous question*) that they need from chemists and shops without being uncomfortable and without sacrificing their privacy and speed (*or whatever is mentioned as an important quality)*.

*Close and thank people for their time*
