# Supplementary material for: Regulating pharmacists as contraception providers: A qualitative study from Coastal Kenya on injectable contraception provision to youth
Source: PLoS One. 2019 Dec 19;14(12):e0226133. doi: 10.1371/journal.pone.0226133 (PMC6922368; doi:10.1371/journal.pone.0226133)
Supplement: S4 File — (PDF) [file pone.0226133.s004.pdf]

Injection Shopper

Please select today's date

yyyy-mm-dd

What time did you enter the chemist?

hh:mm

What time did you exit the chemist?

hh:mm

What is the name of this chemist shop?

- A. Medisink chemist
- A. Destina (Kona ya Msa)
- A. Kona ya Msa chemist
- B. Kona Chief chemist
- B. Jefry chemist
- B. Peace chemist
- B. Hope chemist
- B. Lam chemist
- B. Sellrom chemist
- C. Kaya Chief chemist
- C. Destina chemist 2
- C. Mounristan chemist
- C. Atlas chemist and agrovet
- C. Bien chemist and cosmetic
- C. Mwijo pharmacy
- C. Ochieng 'O' chemist (Ibiza)
- C. Destina chemist (Ibiza)
- C. Umoja chemist
- C. Ibiza chemist
- C. Ultrachem
- C. Sea Eagles chemist (Ibiza)
- D. Sea Side chemist
- D. Southcoast chemist
- D. Sairose chemist
- D. Lola chemist
- D. Sharl chemist
- D. Diani chemist
- E. Sea Eagle chemist (Diani Beach Road)
- E. Ochieng chemist (Diani Beach Road)
- E. Citadel chemist
- E. Salmin chemist
- E. Rachael chemist
- E. Kawawa chemist
- E. Blessis chemist
- E. Alpha chemist
- F. Blessed chemist
- F. Edwardian chemist
- F. Yuspharma
- F. Ukunda chemist
- F. Mwerose chemist
- F. Dawa Point chemist
- G. Point chemist
- G. Harmony chemist
- G. Dawalink chemist
- H. Dyno chemist
- H. Matuta chemist
- I. Konashop 1 chemist
- I. Golini chemist
- I. Konashop 2 chemist
- I. Sea Eagles chemist (Kwale)

Were you offered the chance to buy the injection?

- Yes
- No

If no, why were you refused?

- ○ Didn't have FP card/prescription
- ○ Didn't have injection in stock
- ○ Other

If other, describe:

___

Were you told by the person who helped you that it was possible that HE/SHE could inject you in the shop?

- ○ Yes
- ○ No

If you were told injection in the chemist shop was impossible, what were you told?

- ☐ Person said they were not allowed to give injection themselves
- ☐ Referred to another indivdual qualified to give injections
- ☐ Referred to a clinic/office qualified to give injections
- ☐ Other

If other, describe:

___

Describe the person who helped you and the environment of the chemist

1a. What was their gender?

- ○ Female
- ○ Male

1b. What was their approximate age?

- ○ Aged 20 or younger
- ○ 21-30
- ○ 31-40
- ○ 41-50
- ○ Aged 50+

2. Where in the chemist shop were you helped?

___

3. Did that location provide privacy for your interaction?

- ◯ Yes
- ◯ No

4. Were there other people working in the chemist when you visited?

- ◯ Yes
- ◯ No

5. Were there other customers in the chemist when you visited?

- ◯ Yes
- ◯ No

5b. Do you feel like you had privacy from the other chemists or customers during your interaction?

- ◯ Yes
- ◯ No

Describe the interaction

6. Were you asked any questions about your use of injection?

- ◯ Yes
- ◯ No

7. Did they ask these questions before or after you were offered the injection?

- ◯ before
- ◯ after

» 8. Did they ask:

a. to see your TCA?

- ◯ Yes
- ◯ No

b. whether you had used injection before?

- ◯ Yes
- ◯ No

c. the date of your last injection?

- ◯ Yes
- ◯ No

d. where you received your previous injection

- ◯ Yes
- ◯ No

e. whether you had experienced (or were experiencing) any side effects?

- ◯ Yes
- ◯ No

9. Were you asked any questions about YOURSELF or YOUR HUSBAND?

- ◯ Yes
- ◯ No

9b.Did they ask these questions before or after you were offered the injection?

- ◯ before
- ◯ after

» 10. Did they ask:

a. Your age?

- ◯ Yes
- ◯ No

b. Your marital/relationship status?

- ◯ Yes
- ◯ No

c. Whether you had discussed use with your husband?

- ◯ Yes
- ◯ No

d. Whether you had any children?

- ◯ Yes
- ◯ No

11. Did the person react (do or say something) to any of the information you told him/her?

Did he/she make you feel that something you said affected the way s/he treated you?

- ◯ Yes
- ◯ No

If yes, describe the reaction:

How did he/she react? What did he/she do or say? Were you lectured?

12. Did the chemist give you any information about the injection?

- Yes
- No

12b. Did they give you this information before or after you were offered the injection?

- before
- after

» 13. Did they tell you:

a. instructions on when you needed to return for another injection?

- Yes
- No

b. side effects you might expect?

- Yes
- No

c. that this did not protect you against HIV/STIs?

- Yes
- No

d. Any differences between injection brands?

For example, differences in costs, how long injection lasts, etc

- Yes
- No

Briefly describe any key differences

14. Did they recommend another FP method, or that you go to a health centre?

- Yes
- No

If yes, what method/where were you referred?

Describe the QUALITY of the interaction

15. Approximately how many minutes did you speak with the person who helped you?

16. Did the person who helped you seem like they were in a hurry to finish with you?

Did they seem rushed or give you their undivided attention?

- ◯ Yes
- ◯ No

17. How friendly or approachable was the person who helped you?

1 very unfriendly	2	3 neutral	4	5 very friendly
◯	◯	◯	◯	◯

Briefly describe

18. How knowledgeable did the person who helped you appear to be about family planning?

1 not knowledgeable at all	2	3 average	4	5 extremely knowledgeable
◯	◯	◯	◯	◯

Briefly describe

19. How private was the interaction?

1 not private at all	2	3 neutral	4	5 very private
◯	◯	◯	◯	◯

Briefly describe

20. How comfortable did the person who helped you seem?

1 very uncomfortable	2	3 neutral	4	5 very comfortable
◯	◯	◯	◯	◯

Briefly describe

---

21. Did the person who helped you do or say anything that was particularly helpful or nice?

---

22. Did the person who helped you say or do anything that was particularly NOT helpful or NOT nice?

---

23. How satisfied are you with the overall service?

○ 1 very unsatisfied ○ 2 ○ 3 neutral ○ 4 ○ 5 very satisfied

Briefly describe
